# Pancreatectomy for non-pancreatic malignancies results in improved survival after R0 resection

**DOI:** 10.1186/1477-7819-5-145

**Published:** 2007-12-27

**Authors:** Kimberly A Varker, Peter Muscarella, Kristian Wall, Christopher Ellison, Mark Bloomston

**Affiliations:** 1 Department of Surgery and Division of Surgical Oncology, The Ohio State University Medical Center, Columbus, OH, USA

## Abstract

**Background:**

Pancreatectomy has a high morbidity but remains the only chance of cure for pancreatic cancer. Its efficacy for non-pancreatic malignancies is less clear. We reviewed our experience with pancreatectomy for non-pancreatic malignancies to determine outcomes and identify predictors of survival.

**Patients and methods:**

The records of patients who underwent pancreatectomy for non-pancreatic malignancies between 1990 and 2005 were reviewed. Survival curves were constructed using the Kaplan-Meier method and compared using log-rank analysis. Cox proportional hazards was used to identify predictors of survival.

**Results:**

29 patients (18 M/11 F) with a mean age of 59.9 years (range 29–86) underwent pancreatectomy for non-pancreatic malignancies. 19 (66%) primary malignancies were GI in origin. Most operations were undertaken with curative intent (76%), whereas the remainder was for symptom palliation. Pancreatectomy was completed for metastatic disease in 7 patients (24%) or en bloc to achieve negative margins in 22 patients (76%). Complete (i.e., R0) resection was achieved in 17 (59%). Perioperative mortality was 3%. Median follow-up was 15 months (range 7–172). Median overall survival was 12 months with 1-year survival of 48%. Significant predictors of improved survival by univariate analysis were R0 resection, non-GI primary, and pancreatic metastasectomy (vs. en bloc resection). Only R0 resection was predictive of long-term survival by multivariate analysis (median 21 months vs. 6).

**Conclusion:**

Pancreatic resection for non-pancreatic malignancies can be completed with minimal mortality. However, incomplete resection results in poor overall survival. Pancreatectomy for non-pancreatic malignancies should only be undertaken if complete resection is possible.

## Background

Pancreatic metastases represent an unusual clinical presentation. Sperti *et al*, in a literature review of 2044 reported pancreatic resections, found that only 46 (1.4%) were performed for secondary tumors [1]. Interestingly, the prevalence of metastases to the pancreas at autopsy is reported to be as high as 11%; the majority of these occur in patients with disseminated intraabdominal metastases [2]. Tumors that most commonly metastasize to the pancreas by blood-borne dissemination include renal cell carcinoma, melanoma, and breast and lung carcinomas. Colon carcinoma may involve the pancreas by direct invasion [3]. Up to 50% of patients with metastatic lesions are asymptomatic, the metastasis being detected upon routine surveillance for the primary tumor [3]. The differential diagnosis of metastases to the pancreas as compared to primary pancreatic tumors can be difficult. The presence of multiple hypervascular lesions on imaging studies suggests pancreatic metastasis (whereas primary pancreatic tumors are commonly hypovascular), but primary neuroendocrine tumor of the pancreas must be ruled out [3].

It is well documented that resection of hepatic metastases from colorectal carcinoma, sarcoma, or renal cell carcinoma achieves good survival. Similarly, resections of metastatic disease to the lungs and brain are now routinely undertaken. However, guidelines for pancreatic resection for metastatic disease remain unclear, probably due to the rarity of these tumors. While some recommend resection of pancreatic metastases only when there is no evidence of extrapancreatic disease [4], others maintain that the presence of extrapancreatic disease is not a contraindication, provided that complete resection can be achieved [1,5].

Previous reports of small numbers of patients undergoing pancreatic resection for metastatic lesions have demonstrated median overall survivals of 19–42 months. Selected patients, particularly those with renal cell carcinoma, can achieve long-term survival [1,3,5-11]. These results, along with the decreased morbidity and mortality of pancreatic resection currently obtained in experienced centers, support the aggressive management of pancreatic metastases in selected patients.

We report herein our single-institution experience with 29 pancreatic resections for non-primary lesions of the pancreas, including metastases to the pancreas from previously resected primaries, as well as intraabdominal tumors with direct extension to the pancreas. We sought to determine the morbidity and mortality of pancreatic resection in patients with nonpancreatic malignancies and to identify factors predictive of overall survival.

## Patients and methods

### Patients

The records of patients who underwent pancreatectomy for non-pancreatic malignancies at the Ohio State University Medical Center between 1990 and 2005 (n = 29) were retrospectively reviewed. Both computerized records and paper charts were examined. Approval for this review was obtained from the Institutional Review Board of the Ohio State University.

### Eligibility criteria and patient follow-up

Preoperative evaluation included history and physical examination; laboratory studies including complete blood count, serum chemistry, and coagulation profile; CT of the abdomen; and other imaging studies as appropriate. For patients presenting with pancreatic metastases, records from resection of the primary tumor were also reviewed. Eligibility criteria included the ability to obtain complete resection of the metastatic lesion and/or primary lesion with direct extension to the pancreas, as determined by preoperative imaging assessment; and the absence of medical comorbidities precluding operation. A minority of patients underwent operation with palliative intent. Postoperatively, patients underwent clinical and radiologic evaluation at the discretion of the attending physician.

### Statistical methods

Overall survival was determined from the date of surgery until death from any cause as determined by hospital records or the Social Security Death Index [12] as of May 29, 2007. Survival curves were constructed using the Kaplan-Meier method and comparisons between curves were made using log-rank analysis. All statistical analyses were completed using SPSS v.14.0 software (SPSS, Inc., Chicago, IL).

## Results

### Patients

Between 1990 and 2005, a total of 29 patients (18 male, 11 female; mean age 59.9 years, range 29–86) underwent pancreatectomy for non-pancreatic malignancies at the Ohio State University Medical Center (Table 1). The majority of the operations (76%) were undertaken with curative intent, and the remainder was for symptom palliation. Involvement of the pancreas by direct extension of malignancy occurred in 22 (76%) patients, whereas metachronous metastases to the pancreas from distant sites occurred in 7 (24%) patients (kidney, 4; brain, 1; esophagus, 1; chondrosarcoma, 1). Nineteen primaries (66%) were of GI origin. The most common sites of primary malignancy were colorectal, gastric, and renal cell carcinoma. The majority of patients (21) underwent distal pancreatectomy; 7 underwent pancreaticoduodenectomy and one underwent total pancreatectomy. The median time from primary resection to pancreatic metastasis/extension was 52 months (range, 13–240). Complete (R0) resection was achieved in 17 patients (59%).

**Table 1:** Patient and tumor characteristics.

**Characteristic**	**Number (percent)**
Total patients	29
Gender	
Male	18 (62)
Female	11 (38)
Age	
Median	60.0
Range	29–86
Operative intent	
Curative	22 (76)
Palliative	7 (24)
Indication for pancreatic resection	
En bloc for direct extension of primary malignancy	22 (76)
Metachronous metastasis to pancreas	7(24)
Site of primary tumor	
Colorectal	9 (31.0)
Gastric	8 (27.6)
Renal cell carcinoma	5 (17.2)
Mesenteric fibromatosis	2 (6.9)
Hemangiopericytoma (brain)	1 (3.4)
Breast	1 (3.4)
Esophageal	1 (3.4)
Gallbladder	1 (3.4)
Chondrosarcoma	1 (3.4)
Histologic diagnosis	
Adenocarcinoma	19 (65.5)
Moderately differentiated adenocarcinoma	10 (34.5)
Poorly differentiated adenocarcinoma	5 (17.2)
Mucinous adenocarcinoma	1 (3.4)
Adenocarcinoma not otherwise specified	3 (10.3)
Signet ring	1 (3.4)
Clear cell	5 (17.2)
Mesenteric fibrosis	2 (6.9)
Hemangiopericytoma	1 (3.4)
Chondrosarcoma	1 (3.4)
Extent of pancreatic resection	
Total pancreatectomy	1
Distal pancreatectomy	21
Pancreaticoduodenectomy	7
Completeness of resection	
R0 (complete resection)	17 (59)
R1 (incomplete resection with microscopic disease)	4 (14)
R2 (grossly incomplete resection)	8 (27)

### Morbidity

Operative mortality was 3.4% (one patient). Fifteen patients (51.7%) had a total of 20 complications (Table 2). Overall morbidity in this study was similar to that reported by others [3,13]. The most common GI-related complication was pancreatic fistula (4 patients, 13.8%), followed by intraabdominal abscess and pancreatitis. Pancreatic fistula was defined as drainage of amylase-rich fluid any time after the third postoperative day. The most common non-GI complication was pneumonia (4 patients, 13.8%). Two patients developed deep venous thrombosis. Six patients (20.7%) had complications requiring reoperation. Of these, three patients were explored for small bowel obstructions failing non-operative management. One underwent operation two weeks after pancreatectomy for lysis of adhesions and again at three months for recurrent obstruction, and the other two underwent laparotomy for small bowel obstruction at one and six months postoperative, respectively. One patient underwent revision of gastrojejunostomy at six weeks postoperative, and one underwent exploration for control of gastrointestinal hemorrhage one month after the index operation. Finally, one patient was explored at nine months for small bowel obstruction and was found to have recurrent disease. The incidence of complications among those undergoing R0 resection was not different than that of patients undergoing R1 or R2 resection (*p *= 1, Fisher's exact test). Similarly, the incidence of complications among patients undergoing distal pancreatectomy was not different than that of patients undergoing resection other than distal pancreatectomy (*p *= 0.25, Fisher's exact test).

**Table 2:** Operative complications.

**Event**	**Number (percent)**
Mortality	1 (3.4)
Complications (number of patients)	15 (51.7)
Complications (total number)	20
Pancreatic fistula	4 (13.8)
Intraabdominal abscess	2 (6.9)
Pancreatitis	2 (6.9)
Small bowel perforation with enterocutaneous fistula	1 (3.4)
Anastamotic leak	1 (3.4)
Prolonged ileus	2 (6.9)
Pneumonia	4 (13.8)
Deep vein thrombosis	2 (6.9)
Cerebrovascular accident	1 (3.4)
Wound infection	1 (3.4)
Required reoperation	6 (20.7)

### Survival and prognostic factors

**Figure 1:**
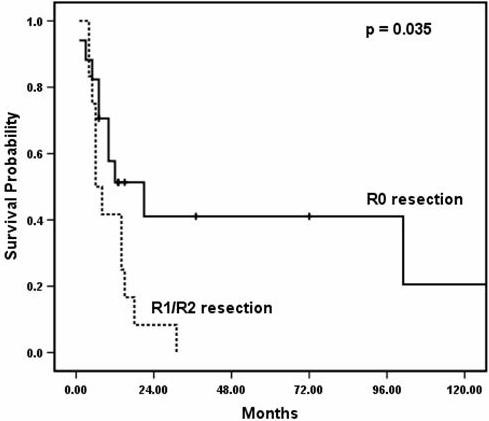
**Survival for R0 resection as compared to R1 or R2 resection**. The solid line represents patients with R0 resection, and the dashed line represents patients with R1 or R2 resection.

**Figure 2:**
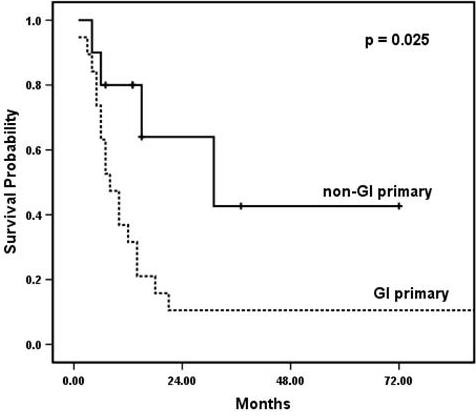
**Survival for gastrointestinal primary as compared to non-gastrointestinal primary**. The solid line represents patients with non-GI primaries, and the dashed line represents patients with GI primaries.

The median length of hospital stay was 12 days (range, 7–43). At a mean follow-up of 14.2 months (range, 1–118), median overall survival was 12 (95% CI 6.4, 17.6) months. 12- and 24-month survival was 48% and 20%, respectively. At the time of analysis, seven patients (24%) were still alive. First, the ability to obtain R0 resection was examined. R0 resection was achieved in 77% of operations undertaken for curative intent, as compared to 0% of operations performed with palliative intent (Table 3). Patients for whom R0 resection was achieved had median overall survival of 21 (95% CI 3.5, 38.5) months, as compared to six (95% CI 3.5, 8.5) months for those who had R1 or R2 resection (*p *= 0.035; Figure 1). Predictors of improved survival by univariate analysis were R0 resection, non-GI primary, and metastasectomy as opposed to en bloc resection (Table 4). By multivariate analysis, only R0 resection was predictive of long-term survival (Table 4). We examined the same set of variables used to evaluate survival (i.e., age, gender, primary site, synchronous versus metachronous disease, disease free interval, curative versus palliative intent, extent of pancreatectomy, metastasectomy versus en bloc resection and R0 versus R1/2 resection) for potential predictors of R0 resection. No factors capable of predicting R0 resection were identified. Next, the primary site was examined. Among patients who underwent en bloc resection for local extension to the pancreas (n = 22), R0 resection was achieved in 12 patients (55%), as compared to 5 of 7 patients (71%) who underwent resection for metastases to the pancreas (Table 3) (*p *= 0.37, Fisher's exact test). Finally, patients with non-GI primaries had improved median overall survival as compared to those with GI primaries: 31 (95% CI 0.0, 61.8) months versus 8 (95% CI 4.6, 11.4) months (*p *= 0.025; Figure 2).

**Table 3:** Likelihood of R0 resection by characteristic (n = 29 patients).

**Characteristic**	**R0**	**R1 or R2**
Intent of resection		
Curative (n = 22)	17 (77%)	5 (23%)
Palliative (n = 7)	0	7 (100%)
Type of resection		
En bloc for direct extension of primary malignancy (n = 22)	12(55%)	10 (45%)
Resection of metastasis to pancreas (n = 7)	5 (71%)	2 (29%)
Site of primary tumor		
Colorectal carcinoma (n = 9)	5 (56%)	4 (44%)
Gastric carcinoma (n = 8)	4 (50%)	4 (50%)
Renal cell carcinoma (n = 5)	3 (60%)	2 (40%)
Other site (n = 7)	5 (71%)	2 (29%)

**Table 4:** Univariate and multivariate analyses for predictors of overall survival. Variables with the greatest potential impact on overall survival by univariate analysis (*p *≤ 0.2) were entered into the multivariate model using Cox Proportional Hazards analysis. Data represent *p *values.

	**Univariate**	**Multivariate**
Age (>60 vs. ≤60)	0.47	--
Age (continuous)	0.52	--
Gender	0.92	--
Primary site	0.20	0.96
Synchronous vs. metachronous	0.14	0.69
Disease free interval (continuous)	0.29	--
Intent (curative vs. palliative)	0.35	--
Pancreatectomy (distal vs. proximal)	0.47	--
Metastasectomy vs. en bloc resection	0.01	0.13
Extent of resection (R0 vs R1/2)	0.04	0.05

## Discussion

Herein we report one of the largest single-institution experiences with pancreatectomy for locally advanced or recurrent non-pancreatic tumors. The majority of these operations were undertaken with curative intent, complete (*i.e*. R0) resection being achieved in 77%. Palliative pancreatectomy, however, did not result in complete tumor clearance in any patient, and was associated with poorer survival. While perioperative mortality was rare (one patient), morbidity was substantial, reflecting the magnitude of the operations performed.

The patient population in this study was somewhat younger than typical patients undergoing pancreatectomy for pancreatic cancer, reflecting our willingness to pursue more aggressive surgical approaches in young, healthy patients. However, age was not a predictor of outcome. The majority of pancreatectomies were performed for cancers of gastrointestinal origin (most commonly colorectal carcinoma), and often for locally advanced disease. Metachronous spread of disease to the pancreas most commonly was of renal origin, as has been demonstrated previously [3,8-11]. Non-gastrointestinal primaries conferred improved overall survival as compared to gastrointestinal primaries, regardless of the completeness of resection.

In this study, we observed a median overall survival of 12 months. This is less than the survival rates reported in previous studies [1,3,5-11]. There are several possible explanations for this finding. Many previous reports of resection of pancreatic metastases include patient populations that were primarily or exclusively composed of patients with renal cell carcinoma. It is well known that metastases from renal cell carcinoma may present at a prolonged interval after resection of the primary, and that long-term survival of up to 10 years after resection of the metastatic lesion is often achieved [8-11]. Although there were five patients in our series with renal cell carcinoma, most patients had GI malignancies, which are generally characterized by shorter disease-free interval and more aggressive tumor biology. In addition, our series included 12 patients (41%) who had R1 or R2 resections.

Several recent reports have demonstrated the utility of resection of colon carcinoma en bloc with pancreaticoduodenectomy for locally advanced disease [14-18]. Kama *et al *detailed the outcome of four patients who underwent en bloc pancreaticoduodenectomy with right hemicolectomy (plus resection of liver segments V and VI in one patient) for locally invasive right colon cancer [14]. There was one postoperative mortality but the remaining three patients had disease-free survival of 14–41 months. Berrospi *et al *studied three patients having en bloc resection for colon cancers involving the pancreas and duodenum; all were alive without evidence of disease at 10, 30, and 113 months, respectively [15]. Similarly, reports by Koea and Curley upon 8 and 12 patients, respectively, undergoing extended resections for colon carcinomas invading the duodenum or pancreatic head revealed low mortality and extended survival [16,17]. Finally, Kapoor *et al *recently evaluated their experience of 11 patients undergoing en bloc resection of adjacent organs for right colon cancer: six had en bloc pancreaticoduodenectomy; three, en bloc local excision of duodenal wall; one, en bloc resection of segments V and VI of the liver; and one, en bloc distal gastrectomy [18]. Median disease-free survival among this group of patients was 54 months. These results suggest that there may be a subgroup of patients with colorectal carcinoma, particularly right colon carcinoma, who have locally invasive disease involving the duodenum or pancreas that responds well to aggressive resection.

Many of the reports in the literature of resection for pancreatic metastases or primaries with extension to the pancreas include only those patients with margin-negative resections. For example, Pingpank *et al*, reported on a population of 35 patients with similar distribution of primary tumor histologies to that reported in this study [19]. However, only patients with negative margins were included. Median overall survival of 46 months was achieved. In contrast, the report by Z'Graggen *et al*, included 6 of 10 patients who underwent palliative procedures only [6]. In this study, a median overall survival of 19 months was observed. Thus, the lower overall survival observed in our study may possibly be explained by both the predominance of patients with GI primaries, and the fact that patients undergoing R1 or R2 resections were included. In order to truly understand the risks and benefits of pancreatectomy for non-pancreatic tumors, we felt it necessary to include all patients undergoing pancreatectomy, including those who had R1 or R2 resection.

As such, we demonstrated that R0 resection, as compared to R1 or R2, in addition to non-GI primary versus GI primary, provided statistically better survival. A univariate analysis showed R0 resection, non-GI primary, and resection of metastatic disease to the pancreas versus en bloc excision for extension to the pancreas to be predictors of survival. However, only R0 resection was predictive of survival by multivariate analysis. This result again emphasizes the fact that complete resection of metastatic or locally advanced lesions must be achieved in order to affect survival.

## Conclusion

Our experience suggests that pancreatic resection for non-pancreatic malignancies can be completed with minimal mortality in experienced centers. This study, albeit relatively small, represents one of the larger experiences to date. Patients with non-GI primary tumors had better survival than those with GI primaries; however, incomplete (R1 or R2) resection resulted in the poorest overall survival, regardless of site of origin. Thus, pancreatectomy for pancreatic metastases or tumors with direct extension to the pancreas should only be undertaken if complete resection is anticipated.

## Competing interests

The author(s) declare that they have no competing interests.

## Authors' contributions

**KV **participated in data acquisition and interpretation, wrote the manuscript, and critically reviewed the manuscript. **PM **participated in data acquisition. **KW **participated in data acquisition (chart review). **ECE **participated in data acquisition and critically reviewed the manuscript. **MB **designed the study, participated in data acquisition and interpretation, and critically reviewed the manuscript. All authors read and approved the final manuscript.
